# Dimension-Factorized Range Migration Algorithm for Regularly Distributed Array Imaging

**DOI:** 10.3390/s17112549

**Published:** 2017-11-05

**Authors:** Qijia Guo, Jie Wang, Tianying Chang, Hong-Liang Cui

**Affiliations:** 1 College of Instrumentation & Electrical Engineering, Jilin University, Changchun 130061, China; gqj2013@gmail.com (Q.G.); wangjie16831@163.com (J.W.); hcui@jlu.edu.cn (H.-L.C.); 2 Institute of Automation, Shandong Academy of Sciences, Jinan 250014, China; 3 Chongqing Institute of Green and Intelligent Technology, Chinese Academy of Sciences, Chongqing 400714, China

**Keywords:** dimension-factorized, range migration algorithm, MIMO, regularly distributed array imaging

## Abstract

The two-dimensional planar MIMO array is a popular approach for millimeter wave imaging applications. As a promising practical alternative, sparse MIMO arrays have been devised to reduce the number of antenna elements and transmitting/receiving channels with predictable and acceptable loss in image quality. In this paper, a high precision three-dimensional imaging algorithm is proposed for MIMO arrays of the regularly distributed type, especially the sparse varieties. Termed the Dimension-Factorized Range Migration Algorithm, the new imaging approach factorizes the conventional MIMO Range Migration Algorithm into multiple operations across the sparse dimensions. The thinner the sparse dimensions of the array, the more efficient the new algorithm will be. Advantages of the proposed approach are demonstrated by comparison with the conventional MIMO Range Migration Algorithm and its non-uniform fast Fourier transform based variant in terms of all the important characteristics of the approaches, especially the anti-noise capability. The computation cost is analyzed as well to evaluate the efficiency quantitatively.

## 1. Introduction

The millimeter wave imager plays an important role in the field of security screening [1,2,3]. In order to build a three-dimensional (3D) image, targets must undergo at least repeated two-dimensional (2D) scans, using, for example, a planar Multi-Input Multi-Output (MIMO) array. To avoid spectral aliasing, which would result in grating lobes, the Nyquist sampling theorem in the space domain must be observed [4]. For MIMO arrays, that means that the transmitting and receiving elements will have to reach a given quantity in a predefined array aperture. This may be impractical in terms of cost or system complexity in some cases. Accordingly, applications and optimization of sparse MIMO arrays have attracted much attention [5,6,7]. 

Two commonly encountered 2D sparse MIMO array scenarios are those with regularly distributed [1,8,9,10] or irregularly distributed elements [7,11]. In the former, the transmitting and receiving elements are arranged evenly across each dimension of the array. Null elements are included, which means that the adjacent elemental interval must be an integral multiple of the minimum interval between elements in each dimension. The latter scenario does not share the same arrangement. The regularity of arrays makes it possible to apply Fast Fourier Transform (FFT) to accelerate the image reconstruction algorithm, but zero-padding will be invoked for the missing elements. 

Extremely high sparsity can be achieved by irregularly distributed MIMO arrays, since the randomness of element layouts tends to share the energy of sidelobes amongst the elements, and the sidelobes and grating lobes will be suppressed according to the projection slice method [12]. Finding approaches to improve the precision of 3D images has been an area of active research focus for irregularly distributed arrays, resulting in a number of methods such as the modified Kirchhoff Migration Algorithm [13], and the grating lobes migration technology [14,15,16]. In terms of efficiency, fast back projection algorithms seem to be attractive [17,18]. In specific cases, these can be as fast as algorithms in the wavenumber domain. They sacrifice precision to achieve acceleration [19,20,21], and sometimes the price may be too high to pay, such as in MIMO array imaging of high sparsity. Precision is critical for a point spread function. To date, a practical fast-imaging algorithm for irregularly distributed MIMO arrays remains elusive.

Any one of the high-precision algorithms mentioned above can be applied to regularly distributed MIMO arrays, and the regularity of MIMO arrays makes it possible to apply the range migration algorithm (RMA), if the performance in terms of efficiency is deemed a priority. 3D MIMO array imaging using RMA was proposed by Zhuge [4], and can, in principle, be applied efficiently to any MIMO array with regularly distributed elements. However, such has not always been the case in practice. The reason lies perhaps in the operation of dimension reduction in the wavenumber domain. Firstly, if the sampling steps in the wavenumber domain across the cross-range dimensions are not equal, a large number of zeroes must be padded for the MIMO array. The performance of RMA will then suffer. This depends on the array geometry, and can be avoided by a proper design. Secondly, even when zero-padding is not needed, the image quality will deteriorate due to the error introduced by the one-dimensional (1D) Stolt interpolation, which will be quite distinct in sparse arrays. A simple remedy to improve the precision is to apply 3D interpolation, but it will be detrimental to the computational efficiency of RMA.

To overcome the aforementioned difficulties, a dimension-factorized range migration algorithm (DF-RMA) is proposed, which exploits the sparsity of some dimensions in the regularly distributed array. MIMO RMA is factorized into multiple RMAs in the sparse dimensions, through which the precision will be enhanced prominently. Two dimensions of the array are sparse generally, and it will not affect the efficiency too much if the sparsity is high enough. As expected, the algorithm will be decelerated as the sparsity is reduced, but the efficiency degradation tends to be limited within a definite boundary. Therefore, the computation load of DF-RMA is still on the same order. On the other hand, the method can be interpreted in the sense of array geometry. The formulation of the whole array is divided into multiple cross-MIMO arrays, which can be formulated very efficiently and precisely by RMA. When combined with parallel computation, the algorithm will be faster.

This paper is organized as follows. The principle of conventional MIMO RMA is reviewed briefly, and DF-RMA is formulated, in Section 2. In Section 3, a numerical simulation is performed to verify the performance of DF-RMA, including the results of a point spread function and imaging of a continuous object in terms of the anti-noise capability. The computation load of DF-RMA is estimated and compared with conventional MIMO RMA. Lastly, the paper will be summarized in Section 4.

## 2. Proposed Method

### 2.1. MIMO Range Migration Algorithm

For MIMO radar architecture, the feature of diversity is a critical advantage for many applications [22], which makes it possible to synthesize a larger aperture with fewer elements compared with phased array architecture. The simplest case of MIMO radar is that with time diversity. The transmitting elements radiate signals in sequence, and the receiving elements receive and record the echoes independently. 

**Figure 1:**
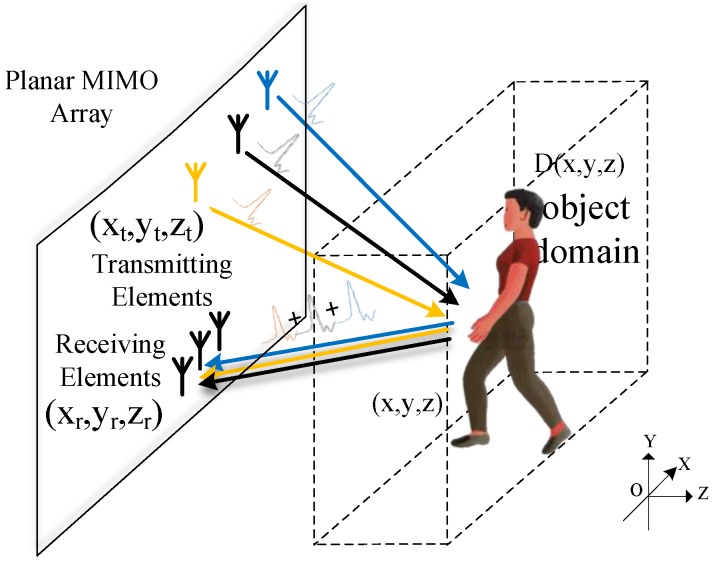
Schematic of a Multi-Input Multi-Output (MIMO) array imaging setup.

The schematic of a MIMO array is illustrated in Figure 1. In Cartesian coordinates, the transmitting and receiving elements form a planar aperture, parallel to the X–Y plane. Targets are supposed to be contained in the object domain D(x,y,z), a block area of Z > 0. Assume that a transmitting element positioned at $\left(x_{t},y_{t},z_{t}\right)$ radiates a pulse to the region Z > 0, and the signal reflected by a scatterer at (x,y,z) is recorded by a receiving element positioned at $\left(x_{r},y_{r},z_{r}\right)$. The plus sign connecting the reflected signals (i.e., the reflection response) represents the principle of superposition among channels. Time division is assumed to be applied here. The echo can be expressed as [23]

(1) $$s\left(x_{t},y_{t},z_{t};x_{r},y_{r},z_{r};k\right)=\underset{D(x,y,z)}{∭}\frac{\sigma \left(x,y,z\right)}{4\pi R_{t}R_{r}}p(k)\exp[-\mathrm{ik}(R_{t}+R_{r})]\mathrm{dxdydz}$$

$$R_{t}=\sqrt{{\left({x-x}_{t}\right)}^{2}+{\left({y-y}_{t}\right)}^{2}+{\left({z-z}_{t}\right)}^{2}}$$

$$R_{r}=\sqrt{{\left({x-x}_{r}\right)}^{2}+{\left({y-y}_{r}\right)}^{2}+{\left({z-z}_{r}\right)}^{2}}$$

k=2πfc

where σ(x,y,z) is the reflectivity function of the scatterer positioned at (x,y,z); f is the frequency of the signal; c is the propagation speed of the electromagnetic wave in free space; p(k) represents the pulse waveform of the signal in the wavenumber domain; and $R_{t}$ and $R_{r}$ denote the single-trip distances from the scatterer to the transmitting and receiving element, respectively.

Computing the 4D Fourier transform on both sides of (1) with the Fourier transform pair $x_{t}\to k_{\mathrm{xt}}$, $y_{t}\to k_{\mathrm{yt}}$, $x_{r}\to k_{\mathrm{xr}}$, $y_{r}\to k_{\mathrm{yr}}$, we get

(2) $$s\left(k_{\mathrm{xt}},k_{\mathrm{yt}},z_{t};k_{\mathrm{xr}},k_{\mathrm{yr}},z_{r};k\right)=-\frac{\pi p(k)}{k_{\mathrm{zt}}k_{\mathrm{zr}}}\underset{D\left(x,y,z\right)}{∭}\sigma \left(x,y,z\right)\;\times \exp[-i(k_{\mathrm{xt}}+k_{\mathrm{xr}})x-i(k_{\mathrm{yt}}+k_{\mathrm{yr}})y]\;\times \exp\left(-{\mathrm{ik}}_{\mathrm{zt}}\left|z-z_{t}\right|-{\mathrm{ik}}_{\mathrm{zr}}\left|z-z_{r}\right|\right)\mathrm{dxdydz},$$

$$k_{\mathrm{zt}}=\sqrt{k^{2}-k_{\mathrm{xt}}^{2}-k_{\mathrm{yt}}^{2}},$$

$$k_{\mathrm{zr}}=\sqrt{k^{2}-k_{\mathrm{xr}}^{2}-k_{\mathrm{yr}}^{2}}.$$

Before further processing, it is imperative to compensate the terms of the free space attenuation and the pulse waveform. Specifically,

(3) $$s_{k}\left(k_{\mathrm{xt}},k_{\mathrm{yt}},z_{t};k_{\mathrm{xr}},k_{\mathrm{yr}},z_{r};k\right)=-\frac{s\left(k_{\mathrm{xt}},k_{\mathrm{yt}},z_{t};k_{\mathrm{xr}},k_{\mathrm{yr}},z_{r};k\right)k_{\mathrm{zt}}k_{\mathrm{zr}}}{\pi p\left(k\right)}\;.$$

According to the matched filtering theory, the reflectivity function σ(x,y,z) can be formulated as [1]

(4) $$\sigma \left(x,y,z\right)=\sum_{z_{t},z_{r}}\int_{D(k)}\iint_{D_{k}{(k}_{\mathrm{xt}},k_{\mathrm{yt}})}\iint_{D_{k}{(k}_{\mathrm{xr}},k_{\mathrm{yr}})}s_{k}\left(k_{\mathrm{xt}},k_{\mathrm{yt}},z_{t};k_{\mathrm{xr}},k_{\mathrm{yr}},z_{r};k\right)\;\times \exp\left[i(k_{\mathrm{xt}}+k_{\mathrm{xr}}\right)x+i(k_{\mathrm{yt}}+k_{\mathrm{yr}})y]\;\times \exp\left({\mathrm{ik}}_{\mathrm{zt}}\left|{z-z}_{t}\right|+{\mathrm{ik}}_{\mathrm{zr}}\left|{z-z}_{r}\right|\right){\mathrm{dk}}_{\mathrm{xr}}{\mathrm{dk}}_{\mathrm{yr}}{\mathrm{dk}}_{\mathrm{xt}}{\mathrm{dk}}_{\mathrm{yt}}\mathrm{dk}.$$

In Equation (4), D(k) is the wavenumber support domain of the detection signal, and $D_{k}(k_{\mathrm{xt}},k_{\mathrm{yt}})$ and $D_{k}(k_{\mathrm{xr}},k_{\mathrm{yr}})$ denote the transmitting and receiving wavenumber support domain, respectively. Assuming $z_{t}=z_{r}\;=\;0$ and z>0, Equation (4) can be reformulated as

(5) $$\sigma \left(x,y,z\right)=\int_{D(k)}\iint_{D_{k}(k_{\mathrm{xt}},k_{\mathrm{yt}})}\iint_{D_{k}(k_{\mathrm{xr}},k_{\mathrm{yr}})}s_{k}\left(k_{\mathrm{xt}},k_{\mathrm{yt}};k_{\mathrm{xr}},k_{\mathrm{yr}};k\right)\;\times \exp\left[i(k_{\mathrm{xt}}+k_{\mathrm{xr}}\right)x+i(k_{\mathrm{yt}}+k_{\mathrm{yr}})y]\;\times \exp\left[i\left(k_{\mathrm{zt}}+k_{\mathrm{zr}}\right)z\right]{\mathrm{dk}}_{\mathrm{xr}}{\mathrm{dk}}_{\mathrm{yr}}{\mathrm{dk}}_{\mathrm{xt}}{\mathrm{dk}}_{\mathrm{yt}}\mathrm{dk}.$$

As expected, RMA ends in the operation of 3D inverse FFT (IFFT). To avoid the effects of the periodicity of Fourier transform, we have to keep the scene center of the image aligned with a predefined distance $H_{c}$, i.e., the center of D(x,y,z) along z-axis. The operation is called scene center compensation, and can be realized by

(6) $$s_{\mathrm{kc}}\left(k_{\mathrm{xt}},k_{\mathrm{yt}};k_{\mathrm{xr}},k_{\mathrm{yr}};k\right)=s_{k}\left(k_{\mathrm{xt}},k_{\mathrm{yt}};k_{\mathrm{xr}},k_{\mathrm{yr}};k\right)\exp\left[i(k_{\mathrm{zt}}+k_{\mathrm{zr}}\right)H_{c}].$$

The term $s_{k}\left(k_{\mathrm{xt}},k_{\mathrm{yt}};k_{\mathrm{xr}},k_{\mathrm{yr}};k\right)$ in Equation (5) shall be replaced by Equation (6). One can also transform Equation (5) into a more compact form as

(7) $$\sigma \left(x,y,z\right)={∭}_{D(k_{x},k_{y},k_{z})}s_{\mathrm{kc}}\left(k_{x},k_{y},k_{z}\right)\mathrm{expi}(k_{x}x+k_{y}y+k_{z}z){\mathrm{dk}}_{x}{\mathrm{dk}}_{y}{\mathrm{dk}}_{z},$$

(8) $$k_{x}=k_{\mathrm{xt}}+k_{\mathrm{xr}},$$

(9) $$k_{y}=k_{\mathrm{yt}}+k_{\mathrm{yr}},$$

(10) $$k_{z}=k_{\mathrm{zt}}+k_{\mathrm{zr}}.$$

In Equation (7), $D(k_{x},k_{y},k_{z})$ is the wavenumber support domain of $k_{x}$, $k_{y}$, and $k_{z}$. Equation (7) is the canonical form of the Fourier transform, which makes it possible to apply FFT. Thus, the 3D image of the reflectivity function σ(x,y,z) will be built after $s_{\mathrm{kc}}\left(k_{\mathrm{xt}},k_{\mathrm{yt}};k_{\mathrm{xr}},k_{\mathrm{yr}};k\right)$ is arranged properly according to Equation (8), (9), and (10). The problem now is how to carry them out efficiently. 

The operations of Equations (8) and (9) are defined as dimension reduction. In Equation (10), $k_{z}$ is nonlinear with k, $k_{x}$, and $k_{y}$. Thus, interpolation must be applied here, which is named Stolt interpolation in conventional RMA. According to [4], Stolt interpolation can be realized by 1D interpolation. Dimension reduction and Stolt interpolation are implemented as follows: Assume the 5D data block $s_{\mathrm{kc}}\left(k_{\mathrm{xt}},k_{\mathrm{yt}};k_{\mathrm{xr}},k_{\mathrm{yr}};k\right)$ is with the size $\left(N_{\mathrm{xt}},N_{\mathrm{yt}},N_{\mathrm{xr}},N_{\mathrm{yr}},N_{r}\right)$ in discrete sample points. The wavenumber sampling steps of $k_{\mathrm{xt}}$ and $k_{\mathrm{xr}}$ must be kept the same by zero-padding if necessary, as must those of $k_{\mathrm{yt}}$ and $k_{\mathrm{yr}}$. The data block will be divided into $N_{\mathrm{xt}}\times N_{\mathrm{yt}}\times N_{\mathrm{xr}}\times N_{\mathrm{yr}}$ vectors with the length $N_{r}$. Suppose the dimensions $k_{\mathrm{yt}}$ and $k_{\mathrm{xr}}$ are sparse; there will be at most $N_{\mathrm{xr}}$ vectors taking up similar $k_{x}$ values, and $N_{\mathrm{yt}}$ for $k_{y}$. These vectors should be reassigned along the dimension k. After dimension reduction, the cross-range wavenumbers turn out to be of the size $\left(N_{\mathrm{xt}}+N_{\mathrm{xr}}-1,N_{\mathrm{yt}}+N_{\mathrm{yr}}-1\right)$, and the number of vectors belonging to the cross-range dimensions will be different. Due to the inconsistency of the range dimension after dimension reduction, 1D interpolation seems to be not precise enough. This problem will not turn up in the case of a single cross array, which is obvious in the example in [2]. A more precise approach is to adopt 3D interpolation, which will inevitably degrade the efficiency. Instead, a nonuniform fast Fourier transform (NUFFT) can be applied here. In the NUFFT-based MIMO RMA, the steps of dimension reduction, Stolt interpolation, and IFFT will be implemented with 3D NUFFT precisely. It would also be more efficient with fast Gaussian gridding acceleration [24]. In the present work, we propose to apply a dimension-factorized range migration algorithm to tackle the task of dimension reduction efficiently without sacrificing precision.

### 2.2. Dimension-Factorized Range Migration Algorithm

**Figure 2:**
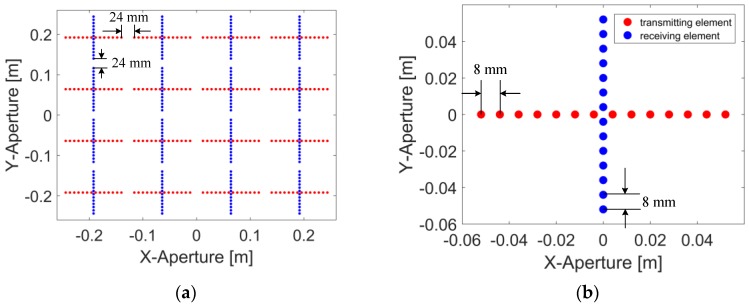
Geometry of the MIMO array: (**a**) Layout of the whole array; (**b**) Unit array.

Our algorithm starts with Equation (7), for a MIMO array with regularly distributed elements that are supposed to be sparse in two dimensions, for instance, a MIMO array geometry as depicted in Figure 2a. The whole array consists of 16 cross-shaped sub-arrays, considered as unit arrays. The whole array can be regarded as the periodic repetition of the unit array. The geometry of the unit array is demonstrated in Figure 2b. The interval between adjacent transmitting (and receiving) elements is 8 mm. There are 14 transmitting elements and 14 receiving elements involved in a single unit array, and 224 transmitting elements and 224 receiving elements totally in the whole array. The gap between the edges of the unit arrays is 24 mm, which corresponds to 2 missing elements. Thus, the cross-range wavenumber $k_{\mathrm{yt}}$ and $k_{\mathrm{xr}}$ represent sparse dimensions, and it will be efficient to compute Equation (5) by factorizing it across the sparse dimensions:

(11) $$\sigma \left(x,y,z\right)=\sum_{n=1}^{N_{\mathrm{xr}}}\sum_{m=1}^{N_{\mathrm{yt}}}\int_{D(k)}\iint_{D_{k}(k_{\mathrm{xt}},k_{\mathrm{yr}})}s_{\mathrm{kc}}\left(k_{\mathrm{xt}},k_{\mathrm{yt}};k_{\mathrm{xr}},k_{\mathrm{yr}};k\right)\;\times \exp\left[i(k_{\mathrm{xt}}+k_{\mathrm{xrn}}\right)x+i(k_{\mathrm{ytm}}+k_{\mathrm{yr}})y+i\left(k_{\mathrm{ztm}}+k_{\mathrm{zrn}}\right)z]{\mathrm{dk}}_{\mathrm{xt}}{\mathrm{dk}}_{\mathrm{yr}}\mathrm{dk},$$

$$k_{\mathrm{ztm}}=\sqrt{k^{2}-k_{\mathrm{xt}}^{2}-k_{\mathrm{ytm}}^{2}}\;,$$

$$k_{\mathrm{zrn}}=\sqrt{k^{2}-k_{\mathrm{xrn}}^{2}-k_{\mathrm{yr}}^{2}}\;.$$

In Equation (11), the wavenumbers $k_{\mathrm{xrn}}$ and $k_{\mathrm{ytm}}$ are the sequenced expressions of $k_{\mathrm{xr}}$ and $k_{\mathrm{yt}}$. The constants $N_{\mathrm{xr}}$ and $N_{\mathrm{yt}}$ indicate the sparsity of the factorized dimensions. In the case represented by Figure 2, we have $N_{\mathrm{xr}}=4$, $N_{\mathrm{yt}}=4$.

In order to elaborate on the computation of Equation (11), it is reformulated as follows:

(12) $$\sigma \left(x,y,z\right)=\sum_{n=1}^{N_{\mathrm{xr}}}\sum_{m=1}^{N_{\mathrm{yt}}}P_{\mathrm{mn}}(x,y,z)\exp({\mathrm{ik}}_{\mathrm{xrn}}x+{\mathrm{ik}}_{\mathrm{ytm}}y),$$

(13) $$P_{\mathrm{mn}}(x,y,z)=\int_{D(k)}\iint_{D_{k}(k_{\mathrm{xt}},k_{\mathrm{yr}})}s_{\mathrm{kc}}\left(k_{\mathrm{xt}},k_{\mathrm{yt}};k_{\mathrm{xr}},k_{\mathrm{yr}};k\right)\;\times \exp[{\mathrm{ik}}_{\mathrm{xt}}x+{\mathrm{ik}}_{\mathrm{yr}}y+i\left(k_{\mathrm{ztm}}+k_{\mathrm{zrn}})z\right]{\mathrm{dk}}_{\mathrm{xt}}{\mathrm{dk}}_{\mathrm{yr}}\mathrm{dk}.$$

**Figure 3:**
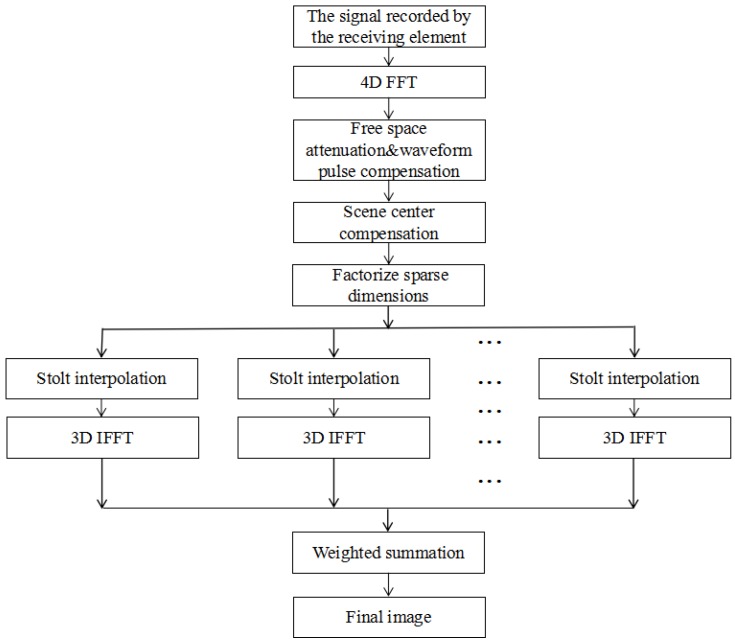
Block diagram of dimension-factorized range migration algorithm (DF-RMA).

Equation (13) can be computed by RMA simply, without zero-padding, or by 3D interpolation. A total ${\mathrm{of}\;N}_{\mathrm{xr}}N_{\mathrm{yt}}$ operations of RMA, i.e.,${\;P}_{\mathrm{mn}}(x,y,z)$, is required independently. As Equation (12) shows, the reflectivity function can be obtained by a weighted summation of $P_{\mathrm{mn}}(x,y,z)$. To summarize the procedure discussed above, Figure 3 presents the block diagram of DF-RMA. Intuitively, the thinner the sparse dimension is, the more efficient our method will be. It seems that the computation load will increase linearly in $N_{\mathrm{xr}}N_{\mathrm{yt}}$, and the efficiency gain of RMA will be lost in the case of very low sparsity. Nevertheless, the computation load estimation will demonstrate quantitatively that the efficiency degradation will not be as bad as expected, in the next section. Moreover, the efficiency can be enhanced once Stolt interpolation and 3D IFFT are computed in parallel.

## 3. Simulation Results and Computation Load Estimation

### 3.1. Point Spread Function

In this section, a numerical simulation is undertaken to demonstrate the validity of DF-RMA and compare it with conventional MIMO RMA and NUFFT-based MIMO RMA. The point spread function (PSF) will be computed first to test the performance in terms of image quality by each algorithm quantitatively.

**Figure 4:**
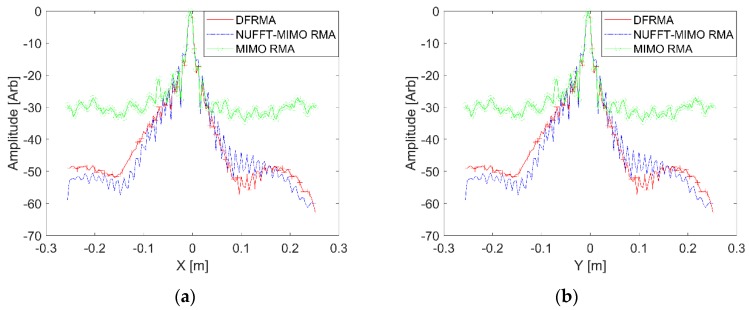
Point spread function (PSF) for each algorithm without noise: (**a**) Profile along *x*-axis; (**b**) Profile along *y*-axis. The red-crossed curve is calculated by DF-RMA, the blue-dotted curve by nonuniform FFT (NUFFT) based MIMO RMA, and the green-circled curve by conventional MIMO RMA.

The PSF is calculated without noise, and the parameters used are listed in Table 1. The geometry of the MIMO array shown in Figure 2 is applied. An ideal scatterer positioned at (0, 0, H_c_) is supposed to be the target, and the echo is calculated with the signal model represented by Equation (1). Three algorithms—DF-RMA, conventional MIMO RMA, and NUFFT-based MIMO RMA—are adopted here to build the 3D image of the scatterer, and the results are presented graphically in Figure 4. These profiles are obtained as follows: The 2D slice corresponding to ${z\;=\;H}_{c}$ is extracted from the 3D image, then the matrix of the slice is projected with the maximum value conserved along the x-axis and y-axis, respectively, so as to present the performance of PSF fairly and comprehensively. As shown in Figure 4, the profiles along the x-axis and y-axis are the same because of the symmetry. For the performance of the full width at half maximum (FWHM) and the maximum sidelobe level (MSL), the difference is not obvious. But for the average background level, it is −45.603 dB in DF-RMA, whereas it is −46.644 dB and −29.710 dB in NUFFT-based MIMO RMA and conventional MIMO RMA, respectively. A suppression gain of about 16 dB has been obtained by DF-RMA with respect to MIMO RMA, and the precision of DF-RMA is as high as that of NUFFT-based MIMO RMA.

### 3.2. Numerical Simulation

**Figure 5:**
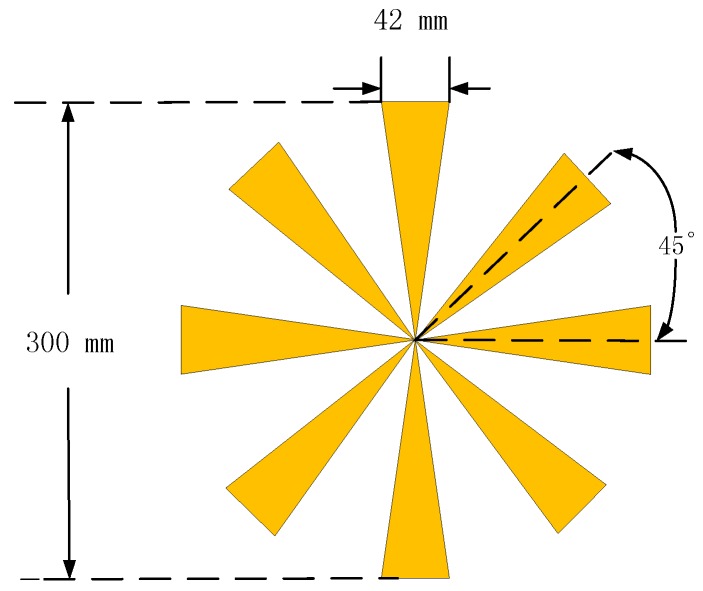
Target: Metal fan-shaped object with eight blades.

In this section, the imaging performance of a contiguous target will be presented as well as anti-noise capability. First of all, the scattering field of the target will be computed by the method of moments (MoM). The target used in the electromagnetic simulation is a perfectly conducting fan-shaped 2D object with eight identical blades, as presented in Figure 5. The geometry of the array in Figure 2 is applied to the simulation, and the other parameters used in this simulation are listed in Table 1. The source used in the electromagnetic simulation is a Hertzian electric dipole. The scattering field is computed at the positions of the receiving elements, and only the co-polarized field will be taken into account. Subsequently, three algorithms are applied to reconstruct the image, and the results are graphically presented in Figure 5. The 3D complex reflectivity function data volume is presented as follows: The pixels are selected based on the maximum absolute values of the data volume in the range direction, and the final image in the cross-range direction is obtained. In this fashion, the mainlobe and maximum sidelobes are retained, and the performance of the algorithms can be compared fairly.

**Figure 6:**
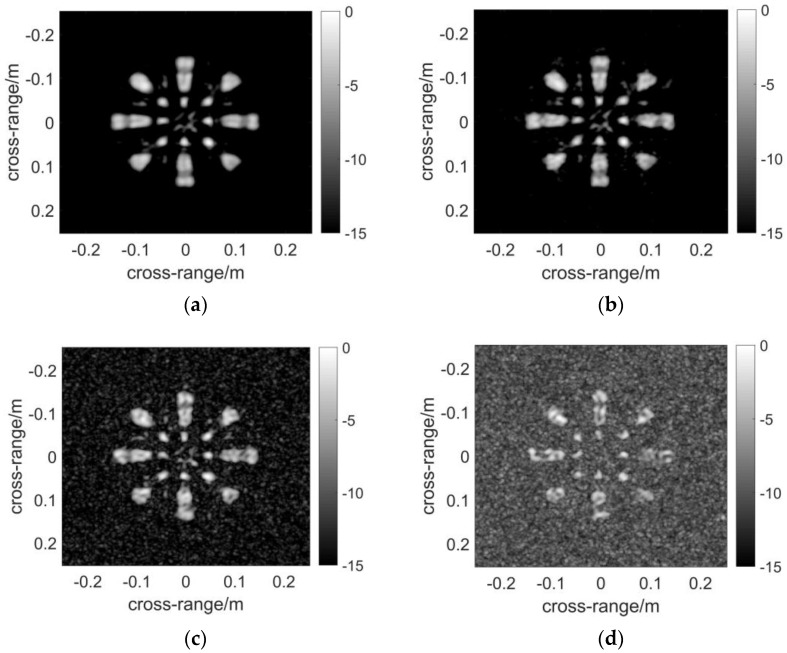
Images constructed using DF-RMA: (**a**) Imaging result without noise; (**b**) Imaging result with signal to noise ratio (SNR) = −15 dB; (**c**) Imaging result with SNR = −20 dB; (**d**) Imaging result with SNR = −25 dB. The pixels of the image are selected based on the maximum absolute values of the data volume in the range direction. The dynamic range of the images is 15 dB.

**Figure 7:**
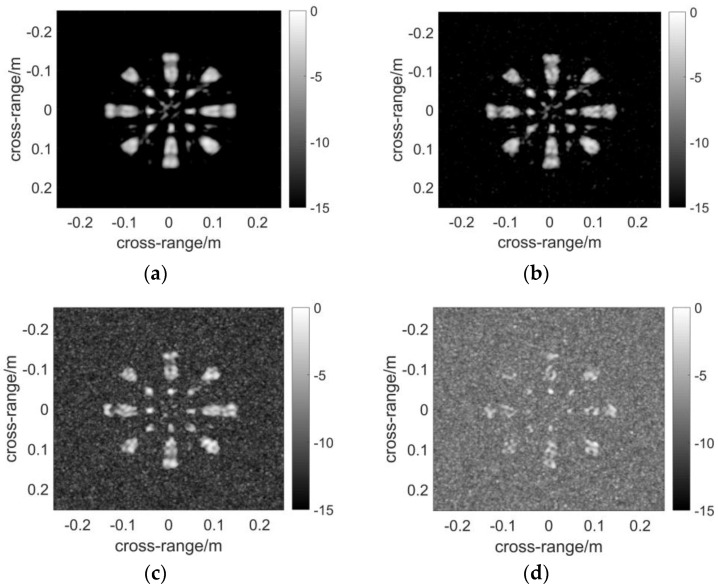
Images constructed using NUFFT-based MIMO RMA: (**a**) Imaging result without noise; (**b**) Imaging result with SNR = −15 dB; (**c**) Imaging result with SNR = −20 dB; (**d**) Imaging result with SNR = −25 dB. The pixels of the image are selected based on the maximum absolute values of the data volume in the range direction. The dynamic range of the images is 15 dB.

**Figure 8:**
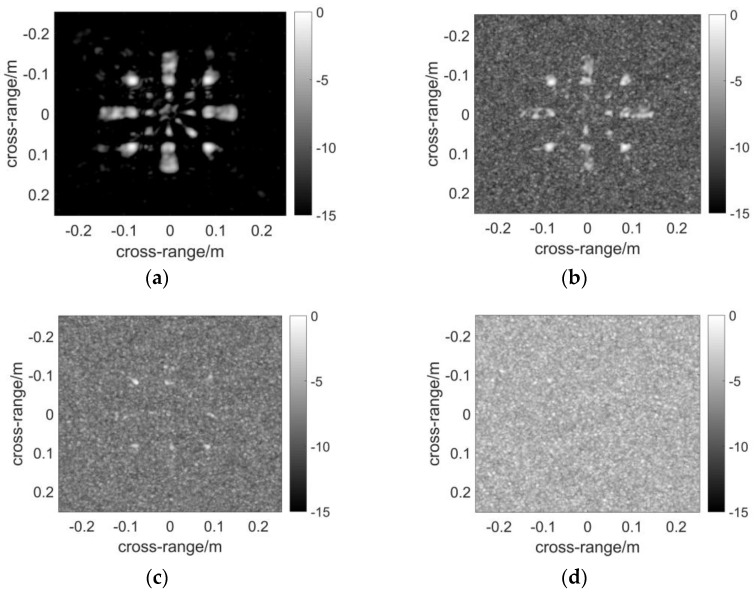
Images constructed using conventional MIMO RMA: (**a**) Imaging result without noise; (**b**) Imaging result with SNR = −15 dB; (**c**) Imaging result with SNR = −20 dB; (**d**) Imaging result with SNR = −25 dB. The pixels of the image are selected based on the maximum absolute values of the data volume in the range direction. The dynamic range of the images is 15 dB.

The resultant Figure 6 is computed by DF-RMA. The profile of the target is rather clear except for the slight grating lobes around the blades without noise in Figure 6a. For sparse MIMO arrays of the regularly distributed types, the grating lobes are unavoidable in the reconstructed image. Subsequently, white Gaussian noise is added to the reflected signal, and the power is measured in terms of the signal to noise ratio (SNR). The result makes no difference when the noise whose SNR equals −15 dB is applied, as demonstrated in Figure 6b. When SNR reduces to −20 dB, the background noise rises and becomes obvious in Figure 6c, but the profile of the target remains recognizable. Noise obscures the resultant image when SNR = −25 dB, which is considered as a critical value of the anti-noise capability of DF-RMA. The result of Figure 7 is computed by NUFFT-based MIMO RMA. The quality of the image is as high as that computed by DF-RMA without noise, and likewise in terms of the anti-noise performance. The result of Figure 8 is computed by conventional MIMO RMA. Even without the added noise, the image in Figure 8a is contaminated by background noise to some extent. Moreover, parts of the scatterer are lost, although the rough sketch of the target remains recognizable. The precision loss by the 1D interpolation is also partially responsible for the image degradation. As noise is added to the signal, the image becomes obscured when SNR = −15 dB in Figure 8b, and the target is buried by noise entirely with lower SNR in Figure 8c,d. In terms of anti-noise performance, DF-RMA is at least 10 dB stronger than conventional MIMO RMA judged by SNR, as the background noise is substantially suppressed by DF-RMA, which is attested by the PSF calculation, too.

### 3.3. Computation Load Estimation

In this section, the computation load of DF-RMA will be evaluated to quantify its deceleration with respect to conventional MIMO RMA. Suppose the constants N_xt_, N_yt_, N_xr_, and N_yr_ are defined as the number of transmitting/receiving sampling points after zero padding along the cross-range directions X and Y, where the subscripts t and r refer to transmitting and receiving elements, respectively. As Equation (1) shows, the echoes are recorded in the frequency domain, and the number of sampling points is N_r_. The requirements of complex multiplications, which constitute the main computational tasks, will be counted to estimate the computation cost.

The computation load of conventional MIMO RMA of each step is listed in Table 2. To balance the efficiency and the precision, 1D piecewise interpolation is an alternative. For each query point, it requires 6 real additions, 2 real divisions, and 2 real multiplications, which is approximated as 1 complex multiplication here. Thus, the computation cost of interpolation is supposed to be $\left(N_{\mathrm{xt}}+N_{\mathrm{xr}}-1\right)\left(N_{\mathrm{yt}}+N_{\mathrm{yr}}-1\right)N_{r}$, just of the same size as the data to be interpolated. It is presumed N_yt_ and N_xr_ are sparse. The wavenumber sampling steps of $k_{\mathrm{xt}}$ and $k_{\mathrm{yr}}$ are supposed to be equal regardless of zero-padding. After dimension reduction, IFFT will be undertaken within the length of $\left({2N}_{\mathrm{xt}},{2N}_{\mathrm{yr}}\right)$ along the cross-range directions. It is convenient to keep the size of output data identical among the algorithms.

In Table 3, the computation load of DF-RMA is evaluated in each step. Differences arise in the last three steps. The algorithm is factorized into $N_{\mathrm{xr}}N_{\mathrm{yt}}$ operations of dimension reduction and IFFT, while the input data have the size $\left(N_{\mathrm{xt}},N_{\mathrm{yr}},N_{r}\right)$ during each operation. Finally, a weighted summation is taken. More computation resources will be required due to these steps. The ratio of the computation load of DF-RMA to that of MIMO RMA is introduced to estimate the efficiency degradation of DF-RMA:

(14) $$\alpha =\frac{T_{\text{DF-RMA}}}{T_{\mathrm{RMA}}}$$

where $T_{\text{DF-RMA}}$ refers to the total computation load of DF-RMA, and $T_{\mathrm{RMA}}$ indicates that of MIMO RMA. To simplify the discussion, the MIMO array is supposed to be symmetric in the cross-range directions, which means $N_{\mathrm{xt}}=N_{\mathrm{yr}}$ and $N_{\mathrm{xr}}=N_{\mathrm{yt}}$ just as depicted by the geometry in Figure 2. According to Table 2 and Table 3, we obtain

(15) $$\alpha_{\mathrm{est}}=\frac{N_{\mathrm{xr}}^{2}\log_{2}\left(N_{\mathrm{xt}}^{2}N_{\mathrm{xr}}^{2}\right)+12N_{\mathrm{xr}}^{2}+4N_{\mathrm{xr}}^{2}\log_{2}(4N_{\mathrm{xt}}^{2}N_{r})}{N_{\mathrm{xr}}^{2}\log_{2}\left(N_{\mathrm{xt}}^{2}N_{\mathrm{xr}}^{2}\right)+2N_{\mathrm{xr}}^{2}+2{\left(\frac{N_{\mathrm{xt}}+N_{\mathrm{xr}}-1}{N_{\mathrm{xt}}}\right)}^{2}+{4\log}_{2}{(4N}_{\mathrm{xt}}^{2}N_{r})}\;.$$

**Figure 9:**
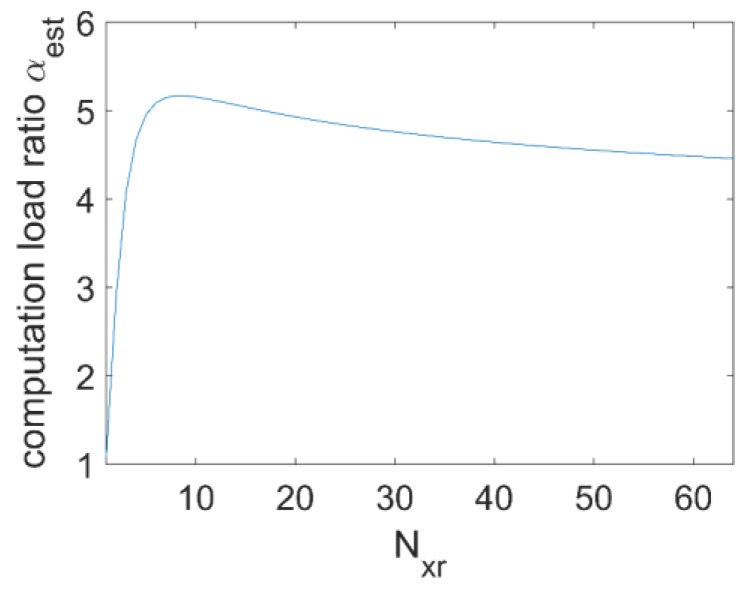
The computation load ratio with respect to N_xr_.

In the numerical simulations of Section 3.1, it is assumed $N_{\mathrm{xt}}=N_{\mathrm{yr}}=64$, $N_{\mathrm{xr}}=N_{\mathrm{yt}}=4$, and $N_{r}=64$. According to Equation (15), we obtain $\alpha_{\mathrm{est}}=4.668$. The programs were run on a personal computer configured with 2-core Intel(R) Xeon(R) CPU i7-6820HQ, and 64 GB RAM. No parallel techniques were applied, and the time consumed by each imaging method is listed in Table 4. The computation load ratio between DF-RMA and MIMO RMA calculated from Table 4 is $\alpha_{\mathrm{meas}}\;=\;4.486$, which is very close to the value $\alpha_{\mathrm{est}}$ estimated from Equation (15). Subsequently, the computation load ratio $\alpha_{\mathrm{est}}$ will be exploited under more severe conditions, when the sparse dimension $N_{\mathrm{xr}}$ varies from 1 to $N_{\mathrm{xt}}$. The extreme case $N_{\mathrm{xr}}=1$ means the MIMO array is a simple cross array, while $N_{\mathrm{xr}}{\;=\;N}_{\mathrm{xt}}$ means no sparse dimensions exist. The plot of the ratio $\alpha_{\mathrm{est}}$ with respect to $N_{\mathrm{xr}}$ is illustrated in Figure 9. We observe that the ratio increases steeply until $N_{\mathrm{xr}}=8$, and thereafter it tends to relax to a stable value around $\alpha_{\mathrm{est}}\;=\;5$. Therefore, the efficiency degradation is limited even when sparse dimensions disappear, and DF-RMA retains the same order of speed under even the worst conditions. Finally, for comparison, we notice that in the case of NUFFT-based MIMO RMA, the efficiency degrades seriously for the implementation of 3D interpolation, even with fast Gaussian gridding.

## 4. Conclusions

In conclusion, a high-precision and efficient imaging algorithm for regularly distributed MIMO array is proposed and demonstrated. Due to the sparse dimensions of arrays, the computation is factorized into multiple operations of RMA. Although the method will be more preferable for arrays with highly sparse dimensions, the efficiency degradation is nevertheless limited under even extremely low sparse dimensions. The salient characteristics of DF-RMA are summarized as follows:

1. In terms of precision, DF-RMA outperforms conventional MIMO RMA significantly. According to a comparison of PSFs, DF-RMA achieves strong background level suppression. In the numerical simulation of a contiguous target without added noise, the quality of the image is better than conventional MIMO RMA, and the precision is as high as NUFFT-based MIMO RMA. As for anti-noise performance, DF-RMA is at least 10 dB stronger than conventional MIMO RMA, judged by SNR;
2. In terms of efficiency, DF-RMA is 5 times slower than conventional MIMO RMA at most (and can be nearly as fast as conventional MIMO RMA in cases of high sparsity), but more than 10 times faster than NUFFT-based MIMO RMA. Equally significantly, the structure of the algorithm is naturally adaptable to parallel computation, and it remains effective in a range of applications, especially those involving sparse arrays.

To summarize, we have proposed and demonstrated a high-precision imaging algorithm with limited efficiency loss, vis-à-vis conventional MIMO RMA for regularly distributed MIMO arrays. It does well in background noise suppression, and outperforms conventional MIMO RMA in terms of anti-noise capability. 

## Figures and Tables

**Table 1:** The parameters in PSF calculation.

Parameters	Value
Operating frequency range	30 GHz–35 GHz
The range from the aperture to the scene center (H_c_)	500 mm
Number of samples in frequency	64

**Table 2:** Computation load of conventional MIMO RMA.

Processing Step	Complex Multiplications
4D FFT	$\frac{1}{2}N_{\mathrm{xt}}N_{\mathrm{yt}}N_{\mathrm{xr}}N_{\mathrm{yr}}N_{r}\log_{2}\left(N_{\mathrm{xt}}N_{\mathrm{yt}}N_{\mathrm{xr}}N_{\mathrm{yr}}\right)$
Scene Center Compensation	$N_{\mathrm{xt}}N_{\mathrm{yt}}N_{\mathrm{xr}}N_{\mathrm{yr}}N_{r}$
Piecewise Interpolation	$\left(N_{\mathrm{xt}}+N_{\mathrm{xr}}-1\right)\left(N_{\mathrm{yt}}+N_{\mathrm{yr}}-1\right)N_{r}$
3D inverse FFT (IFFT)	$2N_{\mathrm{xt}}N_{\mathrm{yr}}N_{r}\log_{2}\left(4N_{\mathrm{xt}}N_{\mathrm{yr}}N_{r}\right)$

**Table 3:** Computation load of conventional DF-RMA.

Processing Step	Complex Multiplications
4D FFT	$\frac{1}{2}N_{\mathrm{xt}}N_{\mathrm{yt}}N_{\mathrm{xr}}N_{\mathrm{yr}}N_{r}\log_{2}\left(N_{\mathrm{xt}}N_{\mathrm{yt}}N_{\mathrm{xr}}N_{\mathrm{yr}}\right)$
Scene Center Compensation	$N_{\mathrm{xt}}N_{\mathrm{yt}}N_{\mathrm{xr}}N_{\mathrm{yr}}N_{r}$
Piecewise Interpolation	$N_{\mathrm{xt}}N_{\mathrm{yt}}N_{\mathrm{xr}}N_{\mathrm{yr}}N_{r}$
3D IFFT	$2N_{\mathrm{xr}}N_{\mathrm{yt}}N_{\mathrm{xt}}N_{\mathrm{yr}}N_{r}\log_{2}\left(4N_{\mathrm{xt}}N_{\mathrm{yr}}N_{r}\right)$
Weight Multiplications	$4N_{\mathrm{xr}}N_{\mathrm{yt}}N_{\mathrm{xt}}N_{\mathrm{yr}}N_{r}$

**Table 4:** Time consumed by each imaging method.

Method	Time Consumed (s)
DF-RMA	10.243
NUFFT based MIMO RMA	112.838
Conventional MIMO RMA	2.283
